# The Impact of the “Semente” Program on the Family-Focused Practice of Mental Health Professionals in Portugal

**DOI:** 10.3389/fpsyt.2019.00305

**Published:** 2019-05-07

**Authors:** Karin van Doesum, Teresa Maia, Catarina Pereira, Monica Loureiro, Joana Marau, Lurdes Toscano, Camilla Lauritzen, Charlotte Reedtz

**Affiliations:** ^1^Regional Centre for Child and Youth Mental Health and Child Welfare, Arctic University of Norway, Tromsø, Norway; ^2^Department of Clinical Psychology, Radboud University, Nijmegen, Netherlands; ^3^Mental Health Department, Hospital Prof. Doutor Fernando Fonseca, Amadora, Portugal

**Keywords:** family focused, mental illness, parenting, implementation, children of parents with a mental illness (COPMI)

## Abstract

**Background:** Children of parents with mental illness (COPMI) are a high-risk group. There is a strong association between parents’ psychiatric disorders and the incidence of psychopathology in their children. These children need to be identified and supported by mental health workers early, and hence, clinical practice in adult mental health services needs to change from a focus on individual patients to a more systemic family focus. “Semente,” a mental health promotion program developed by the Psychiatry Service of Fernando Fonseca hospital (Lisbon, Portugal), had been established to identify these children and families and promote their mental health, by decreasing the impact of risk factors and promoting protective factors. The program included preventive COPMI interventions and implementation of activities offered to families with children in the mental health care. The aim of the present study was to evaluate changes in mental health care after the training in “Child Talks” intervention (two to three psycho-educational meetings with parents and children) and implementation of the “Semente” program.

**Methods:** Participants (*N* = 51) were all professionals from Psychiatric Service of Fernando Fonseca Hospital who received *Child Talks* training. The Family-Focused Mental Health Practice Questionnaire (FFMPQ) was used to measure change in professionals’ attitudes, knowledge, confidence, and organizational structure in working with these families. All participants filled in the questionnaire before training and 10 months later.

**Results:** The results showed that, from pre- to post-measurement, the professionals changed clinical practice significantly. The largest changes were visible in the improved provision of support at the workplace for family-focused practice and the clarity and availability of the policies and procedures. Furthermore, the skill and knowledge of the mental health workers showed significant improvement at posttest.

**Conclusion:** The positive results of this study were not unexpected; the training, implementation of routines, and procedures as well as workplace support were aims of the “Semente” program. Interpretation of the results should be taken with caution because of the small sample and the lower reliability of some of scales of the FFMPQ. The results indicate that professionals moved from patient focus to family focus during the implementation of the “Semente” program.

## Introduction

Children of parents with mental illness (COPMI) are a high-risk group in society. About 15–23% of children live with a parent with a mental illness worldwide (1, 2). Recent research has demonstrated a strong association between parental psychiatric disease and a higher psychiatric risk in their children (3, 4). About one-third of these children experience serious mental health problems when they grow up, and another one-third experience more serious and long-term adjustment issues (2, 5). To illustrate, a prospective cohort study of 256 children who have a parent with depression or anxiety showed that 38% had developed an affective or anxiety disorder by age 20. At 35 years old, about 65% had depression or an anxiety disorder (6).

In the last decades, many studies showed adverse outcomes in children of parents suffering from mental illness, including negative effects on children’s social, behavioral, emotional, and cognitive development (4, 7–9). Moreover, school failure and deficits of social competence at a young age are very common (10–12), sometimes followed by poor functioning at work and in intimate relationships later in life (13) and are at higher risk for suicidal behavior (14).

Mental health stigma and social isolation of these families have contributed to the lack of attention to these children. It is also a population with high genetic and social risk, namely, transmission of mental disorders from parents to children, inheritance of social environment framework of the family, neglect, child abuse, and dysfunctional family interactions associated with the parental psychopathology (3, 8, 15, 16). These children and adolescents frequently suffer in silence from the problems of their parents’ mental illness. Several studies have demonstrated that they often experience social isolation. Their social contacts could be limited because of their parents’ poor functioning. Frequently, the child takes over a parental role in the family. On the other hand, stigma that involves their parents’ mental illness can create negative feelings, such as shame and guilt. Because of that, these children tend to not communicate about their emotional experiences at home (17).

Given this scenario, it becomes evident that preventive intervention is clearly needed to minimize the adverse effects of having parents with mental illness. In their review on interventions to prevent emotional problems in COPMI in the Netherlands, Van Doesum and Hosman (18) postulated that many of the identified risk and protective factors may be sensitive to modulation following exposure to preventive interventions. They pointed to a range of interventions that focus specifically on parents and entire families: making them aware of the impact of the home situation for the children, supporting and informing them, improving the quality of parent–child interaction, and facilitating external social support for children and parents. In Australia and several countries in Europe, prevention programs for children of parents with a mental illness have been developed and are considered a high priority in public mental health policy. Internationally, there is increasing evidence demonstrating the effectiveness of a number of interventions to prevent COPMI from developing problems themselves [e.g., Refs. (7, 19–21)]. However, implementing such interventions requires a systematic approach to changing the behavior of adult mental health service practitioners (22). Children of mentally ill parents need to be identified and supported in adult mental health care in an early phase, and hence, clinical practice in adult mental health services needs to change from a patient focus to a family focus.

However, implementing changes in the practice of adult mental health care to facilitate identification and support of children of mentally ill patients can be challenging, and this large population of high-risk children remains overlooked. There is a lack of natural or systematic communication between psychiatrists treating ill patients and child psychiatry services. Professionals who are treating the parents do not often inquire about the development and mental health of their children. Similarly, pediatric psychiatric teams rarely work with parents’ clinicians, even though both groups of mental health professionals are aware that chronic mental disorders burden families (23).

Nevertheless, according to a systematic literature review by Siegenthaler and colleagues (24), interventions to prevent mental disorders or psychological symptoms in COPMI appear to be effective and decrease the risk of developing the same mental illness as the parent by 40%. A current meta-analysis of Thanhauser and colleagues (25) confirmed significant but small effects on preventive interventions for COPMI. They found small significant effect sizes enhancing mother–infant interaction and small effects on global child’s psychopathology as well as internalizing problems in adolescents. Interventions addressing parents and children together produced overall larger effects. Research on family-focused practice showed that recognition of the benefits of including families in mental health care is increasingly growing (26–28). It is being reflected in governments’ policies and standards, and these interventions are an effective response to these families when collaboration with a range of service sectors and professions is included (26, 29, 30). A continuum of family-focused activities for mental health professionals is recommended when working with parents with mental illness, ranging from assessing their parenting competence to providing appropriate information and resources to family members to providing a liaison with other services to give the family support as needed (31). In a recent meta-analysis, Foster and colleagues (26) identified six core, interrelated family-focused practices: assessment; psychoeducation; instrumental, emotional, and social support; family care planning and goal setting; as well as liaison between families and services. They found that the most commonly described feature of family-focused practices in mental health services was psychoeducation. According to Reupert and Maybery (32), it is essential to develop the most appropriate interventions. They argue that COPMI may be educated about their parent’s mental illness through bibliotherapy, peer support groups, and individual consultations with mental health professionals and family consultations with mental health professionals.

It is clear that receiving psycho-education about the parent’s mental disorder, understanding the reasons for changes in behavior, and knowing and talking about what is happening within the family is important for children as well as for the parents (7, 27, 33). Communication in the family not only encourages family problem solving but also has been proven to build both child and family resilience. One of the interventions focused at the family is *Child Talks*, developed in the Netherlands (18, 34). It is a brief intervention that was developed so professionals can offer support when their patient has children. Adult mental health workers are trained in talking to parents and children, and they offer this intervention as part of the treatment (34). *Child Talks* consists of three conversations with the parents and the children together and focuses on children in the family, parental concerns, and how parental mental health problems may have an impact on children. It also includes a talk with the children and parents together. It is designed as a basic offer for all parents with mental illness and/or substance abuse and can be used as a standard procedure to talk with parents and children in the adult mental health care. When a parent is registered in the adult mental health care, *Child Talks* is offered during the intake meeting by the therapist of the parent or by a child responsible person specially appointed to talk to the family. The family sessions are offered at the clinic or during home visits. *Child Talks* is implemented and adopted in northern Norway (16). Regarding this intervention, Reedtz and Lauritzen (35) found in a retrospective study of electronic patient journals in the University hospital in north Norway that implementing *Child Talks* in adult mental healthcare may lead to clear identification of patients who are parents and more referrals of children in need of more extensive interventions (*N* = 5,268 in 2010, *N* = 5,599 in 2011, and *N* = 5,705 in 2012).

Nonetheless, the implementation of these interventions requires a great deal of involvement of professionals. Lauritzen and her colleagues (36) evaluated the factors that may facilitate or hinder a family focus in the treatment of parents with mental illness in Norway in a sample of 219 professionals, and concluded that professionals with additional training in family-focused approaches to treatment, such as *Child Talks*, are found to be more open to and active in pursuing a child-focused clinical practice. To evaluate the professionals’ perceptions about their own practice, Maybery et al. (37) designed the *Family Focused Mental Health Practice Questionnaire (FFMHPQ)*. The authors compared different groups of professionals in terms of their use of family-focused practices. They concluded that “where more intensive intervention is required for these families, the professionals involved will need training in relation to the evidence-based interventions that are known to be effective with this target group.” Given this, it is recommended that all adult mental health professionals should receive workplace training in basic family-focused practice (30).

### The Semente Program

“Semente” is a mental health promotion program developed by the Psychiatry Service of Fernando Fonseca Hospital, a general hospital in the suburbs of Lisbon (Portugal). It was established with the financial support of EEA grants,^1^ with the objective of identifying COPMI and supporting them with preventive interventions. The program is intended to implement new practices focused on prevention, and targets children and families with parental psychopathology. Being responsible for the treatment of patients with mental disorders, the Adult Psychiatry professionals have easy access to these children through their parents who are also patients.

More specifically, the “Semente” program aims to promote children’s mental health and prevent the development of later mental health problems in this vulnerable group, by early identification of these children and improving parental competencies in parents with psychiatric disorders, as well as providing treatment of psychiatric symptoms in the children by the Child Adolescent Psychiatry service, when necessary.

The development of “Semente” was based on the organizational model of the Psychiatry Service, which has a main focus on community interventions, and was made possible through the establishment of partnerships with primary health care, public health, the municipalities, child protection services, schools, and several NGOs. This Service is organized in a community-based model to improve the access of patients with mental disorders, reduce stigma, and reinforce support networks for the patients and their families. The service integrates four community multidisciplinary mental health teams, responsible for the treatment of adult patients—mainly those with severe psychiatric disorders—and a Child and Adolescent Psychiatric Unit. There is a close relation and communication between the child and the adult psychiatry teams, which coordinate their work with complex families, through monthly meetings and regular case discussion and supervision.

“Semente” has focused on making mental health workers aware of the vulnerability of COPMI and has trained professionals in a new approach involving the children of patients with a mental illness as an issue in their daily practice. Training on COPMI preventive interventions focused on their needs is of great value to guarantee further implementation of this program, as well as the accuracy of structured interventions. The chosen approach includes the family-focused preventive intervention *Child Talks*. The choice of this intervention was based on reflections on how to reconcile preventive interventions with the great demands of clinical practice in order not to overload professionals. All the professionals from the Adult Mental Health Community teams and from Child and Adolescent Psychiatry team were trained in the *Child Talks* intervention according to the training model developed in the Norwegian project by Reedtz and colleagues (16). The training consists of 1.5 days of lectures and role playing concerning how to talk to parents and children, as well as discussions about barriers and possible solutions.

### The Current Study

The overall aim of the present study was to evaluate changes in the self-perception that clinicians have about their clinical practice after the training in basic family-focused practice *Child Talks*. The following research questions were posed: 1) Will training in *Child Talks* change a patient-centered focus into a family focus in adult mental health practice? 2) Will training in *Child Talks* change professional attitudes towards working with patients who are parents? 3) Will training in *Child Talks* improve organizational policy and support? 4) Will training in *Child Talks* improve workers skills and knowledge in working with the whole family?

## Method

### Participants

The participants in the study are the professionals in the adult and child psychiatric facility at Fernando Fonseca Hospital, since these two groups of professionals were involved in the program in order to better evaluate and respond to the needs of all patients’ family members.

At pre-measures, 51 mental health workers responded, corresponding to 90% of the total staff (12% were child providers). As described in **Table 1**, the respondents were mostly women (82% of the cases), and their age ranged between 24 and 56 years old. At post-measures, after the training of *Child Talks*, 46 professionals responded, with essentially the same gender ratio (85% women) and age range (between 25 and 57 years old) as the full sample. The retention rate was 90%. Professionals from both adult and child psychiatry participated at both measurement points. The study was approved by the Ethical Health Commission of the Hospital Prof. Doutor Fernando Fonseca, ensuring a good clinical practice.

**Table 1:** Sociodemographic characteristics of participants at pre- and post-measurement.

	Pre (*N* = 51)	Post (*N* = 46)
**Gender**		
Women	42	39
Men	9	7
**Mean age**		
Women	42	43
Men	34	35
**Adult psychiatry**	45	40
**Child psychiatry**	6	6
**Profession**		
Social worker	5	5
Nurse	10	9
Psychiatry resident	12	12
Psychologist	7	6
Psychomotor therapist	3	3
Psychiatrist	10	8
Child psychiatrist	1	–
Occupational therapist	3	3

### Materials

**Sociodemographic characteristics.** A short questionnaire was developed to collect information on gender, age, and profession.

**Family Focused Mental Health Practice Questionnaire (FFMHPQ).** This questionnaire was used to measure changes in training needs, interests, and attitudes regarding family-focused practice over a period of time. The scale is designed and tested by Maybery and colleagues (37). Family-focused mental health practice is defined by how professionals involve the whole family in treatment and examines the parenting role of the patient. The questionnaire includes 49 items and 16 subscales with statements relating to professionals’ knowledge and skills about family issues; their interest in working with children, parents, and families; their perception of organizational policy and support for family-sensitive practice; and their level of family-focused practice undertaken in their work. **Table 2** shows the questionnaire’s subscales, with definitions and examples of items. Each item is rated on a seven-point Likert scale (ranging from *strongly disagree* to *strongly agree*). A low score on the subscales suggests a low degree of family focus, and a high score shows high degree of family focus. The measure has a good content and construct validity and generally fair internal subscale reliability (38). The questionnaire was used in several samples with variety of professional disciplines in the mental health care in Australia (e.g., 30) and in a sample of Irish mental health nurses (39). The questionnaire was translated into Portuguese by the project team and back into English to ensure good reliability. Cronbach alphas were computed for all subscales and range from .42 to .81 (see **Table 2**). The subscale “local issues” had an unsatisfactory reliability of Cronbach’s α = .26 and was hence excluded in the results.

**Table 2:** Subscales of the FFMHPQ, definition and item examples (retrieved from 37).

Cronbach’s alpha	Subscale	Definition	Example item
.66	Workplace support (2 items)	*The workplace provides support for family-focused practice*	My workplace provides supervision and/or mentoring to support workers undertaking child-related work in regard to their consumer-parents
.78	Time and workload (3 items)	*Time or workload issues regarding family-focused practice *	There is no time to work with families or children
.50	Policy and procedure (2 items)	*Family focused policy and practices are clear at the workplace *	Government policy regarding family-focused practice is very clear
.53	Professional development (2 items)	*There are opportunities for professional development regarding working with families *	My workplace provides little support for further training in family-focused practices
.42	Coworker support (2 items)	*The support from other workers regarding family-focused work *	I often receive support from co-workers in regard to family-focused practice
.47	Family and parenting support (5 items)	*Providing resources and referral information to consumers and their families *	I regularly provide information (including written materials) about mental health issues to the children of consumer-parents
.53	Work confidence (3 items)	*The level of confidence the worker has in working with families, parents and children*	I am not confident working with consumer-parents about their parenting skills
.71	Support to carers and children (2 items)	*The level of information, advocacy and referral provided to carers and children*	Rarely do I advocate for the carers and/or family when communicating with other professionals regarding the consumer-parent’s mental illnesses
.54	Engagement issues (3 items)	*The opportunity for engagement with family members *	Discussing issues for the consumer parent with others (including family) would breach their confidentiality
.79	Assessing impact on child (2 items)	*How well the worker assesses the impact of the parent illness on the children *	I am able to determine the developmental progress of the children of my consumer-parents
.81	Training (4 items)	*Worker willing to undertake further training*	I would like to undertake future training to increase my skills and knowledge for working with the children of consumer-parents
76	Skill and knowledge (5 items)	*Worker skill and knowledge regarding impact of parental mental illness on children*	I am knowledgeable about how parental mental illness impacts on children and families
.44	Service availability (2 items)	*There are programs to refer families to*	There are no parent-related programs (e.g., parenting skills) to refer consumer-parents to
.69	Connectedness (3 items)	*Workers assessment of parent awareness of child connectedness*	I am able to determine the level of importance that consumer-parents place on their children maintaining strong relationships with other family members
.58	Referrals (2 items)	*Referring family members to other programs *	I refer consumer-parents to parent-related programs (e.g., parenting skills)
.52	Interdisciplinary cooperation (4 items)	*Workers possibility to cooperate with other professionals *	Children and families ultimately benefit if health professionals work together to solve the family’s problems

FFMHPQ, Family Focused Mental Health Practice Questionnaire.

### Procedure

**Training.** In July 2015, the whole staff was trained in the *Child Talks* intervention by a trainer from the Netherlands; a supervision meeting with the trainer followed in November. After the training, everyone was able to use *Child Talks* in their practice. The evaluation of the training was carried out through the administration of a qualitative questionnaire, where questions were asked about the global satisfaction with the training, the quality of the content, the structure and the organization of the training, the clarity of the objectives, theoretical background and aspects of application of the intervention, some aspects of motivation to use *Child Talks*, perceived confidence in working with children and families, and overall opinion of this intervention. The participants answered on a scale from 1 (Very dissatisfied) to 5 (Very Satisfied), and responses ranged from 4 to 5—participants considered the training overall very well organized, useful, a way of increasing competencies in relation to COPMI, and very good initiative to improve communication between parents and children, and also indicated that the management supported implementing *Child Talks* in the future.

**Practicing *Child Talks*.** The patients with children under 18 years old were gradually identified during regular consultation at community mental health teams. An administration form was developed so that mental health workers could identify these families, and refer them to *Child Talks* intervention. During interventions, the professionals filled a logbook of the family meetings. Although *Child Talks* was not initially designed to be delivered by two mental health workers, the project team decided that their meetings with parents and children should be done by two persons, one of them the professional being the professional who regularly provides treatment to the patient. Between two and three sessions were offered to the families who agreed to participate. The fidelity was checked by a supervision meeting with the trainer, and all cases were discussed as part of the monthly staff meetings in the psychiatry service of hospital Fernando Fonseca.

**Measurements.** The FFMHPQ was distributed by the coordinating team of “Semente” before the training on *Child Talks* (pre-test) to all the mental health professionals of the Psychiatry Service. The second measurement was performed 10 months later when *Child Talks* was already implemented in the mental health service (Posttest).

### Data Analysis

All statistical analyses were performed with SPSS (version 25). Descriptive analyses were used to explore the demographic details of the groups. A dependent sample t-test was used to test the difference between the pre- and post-implementation phase. Cohen’s *d* was calculated to express effect sizes. A Cohen’s *d* = .80 is a large effect, *d* = .50 is a medium effect, and *d* = .30 is a small effect.

## Results

### Change in Family Focus

In general, the results indicated that, from pre to post, the professionals of the mental health care in the Fonseca Hospital changed their perception about clinical practice significantly by increasing their competencies and moving their focus to the children of their patients (see **Table 3**). Overall, medium to large changes were seen in 11 of the 16 scales of FFMHQ.

**Table 3:** Paired t-tests of difference in outcome of subscales FFMHPQ between pre- and post-training.

	Pre-test		Post-test		*t*	Cohen’s *d*
	*N* = 51		*N* = 46			
	*M*	*SD*	*M*	*SD*		
**1. Organizational policy and supports**						
- Workplace support	4.37	1.30	5.32	1.04	−4.07***	0.81
- Time and workload	3.94	1.42	3.63	1.22	0.60	0.31
- Policy and procedure	2.91	1.05	3.76	1.10	−4.63***	0.79
- Professional development	5.08	1.23	5.64	0.99	−2.36*	0.50
- Coworker support	5.04	1.01	5.11	0.88	−0.30	0.07
**2. Working with patients who are parents**						
- Family and parenting support	3.29	0.79	3.69	0.86	−2.66**	0.49
- Work confidence	4.31	1.04	4.77	0.93	−2.49*	0.46
- Support to carers and children	5.29	0.85	5.44	0.79	−0.91	0.18
- Engagement issues	4.41	0.80	4.40	0.70	0.28	0.01
**3. Worker skill and knowledge**						
- Assessing impact on child	4.11	1.37	4.75	1.20	−2.46*	0.50
- Training	6.07	0.68	5.64	0.85	2.54*	0.55
- Skill and knowledge	4.00	1.06	4.90	0.92	−4.86***	0.90
- Service availability	3.52	1.20	4.16	1.27	−2.10*	0.52
- Connectedness	4.82	0.97	5.12	0.91	−1.88	0.32
- Referrals	3.26	1.33	4.00	1.19	−2.63*	0.59
- Interdisciplinary cooperation	6.26	0.57	5.98	0.58	2.09*	0.49

*p < .05; **p < .01; ***p < .001 (two-tailed test).

### Professional Attitudes Working With Patients Who Are Parents

Results showed moderate positive changes (Cohen’s *d* between .49 and .59) in the professionals’ perception of their clinical practice regarding the way they address family and parenting, and in their self-confidence in working with the whole family. No changes were visible in the engagement with family members, neither in support to caregivers, children, nor other engagement issues.

### Organizational Policy and Support

The organizational policy and support improved significantly, especially the provision of support in the workplace related to family-focused practice (like supervision), as well as the clarity in and availability of policy and procedures (Cohen’s *d* between .79 and .81). A smaller change was found in the opportunity for professional development regarding working with families (Cohen’s *d* = .50). No changes were found in time available, workload, or coworker support.

### Worker Skill and Knowledge

Skills and knowledge of the mental health workers related to the impact of parental mental illness on children improved significantly at posttest (Cohen’s *d* = .90). Moderate positive changes were found in the improvement of the mental health workers’ ability to assess the impact of the parental illness on the children; the professionals believe in making services more available and that they increased the number of referrals Cohen’s *d* between.49 and.59. For interdisciplinary cooperation and training, both subscales declined significantly at posttest. The means of these subscales were already high both in pre- and post-training—both areas most valued by professionals, with the highest scores for all the subscales in post-test. No effect was found in the importance of parents’ awareness of child connectedness, e.g., relationships with family members and friends.

## Discussion

The findings of this study showed that the self-perception about clinical practice in the mental health care of the participating hospital changed from pre- to post-measurement. During this time, professional received training in *Child Talks* and implemented new routines. Consecutively, we found evidence of change in each of the four research questions. 1) Based on the overall improvement in family-focused practice after 10 months, we interpret the findings of the study such that the patient focus of the professionals changed into an approach where they focused more on the whole family. 2) Furthermore, the improvement of professionals’ self-confidence in working with the whole family is evident in the professional attitudes in working with patients who are parents, and these attitudes changed in a way where they address family and parenting to a higher degree after training and implementation. 3) The organizational policy and supports showed improvement as the provision of support for family-focused practice in the workplace increased. Also, the clarity and availability of policy and procedures, and the opportunity for professional development regarding working with families were strengthened. 4) Worker skill and knowledge improved as the awareness about impact of parental mental illness on their children and the way to assess this impact on the children increased. Furthermore, the professionals started believing that they could make services more available and that they could increase the number of referrals. According to interdisciplinary cooperation and training, both subscales significantly declined at posttest, showing that there was less need for training and less need for interdisciplinary cooperation.

Although the results of the study are mainly positive, one should be careful with the interpretation of the results. First, the results of this study were not unexpected, as the training and implementation of new routines and procedures, as well as provision of workplace support, were the aims of the “Semente” project. As extra efforts (time, training, and finance) were available during the 10-month period, this was not surprising. Even though some large changes were found in organizational policy and professionals’ skills and knowledge, the changes in clinical practice and the actual work with children and parenting perspectives in patients were smaller. The question is whether the changes in clinicians are sufficient and sustainable over time. The short-term changes in the project can be seen as the result of initial implementation stage, but long-term changes in full operational stages in the innovative project will also need to be evaluated (40). Therefore, the sustainability of the implementation needs to be monitored in the subsequent years. The stage where new interventions are installed and put into use by all staff as a regular service is the most challenging stage of implementation, and according to implementation theories and experiences, it requires 2 to 4 years’ time (40). The reasons for this are multifaceted, but for example, skilled mental health workers and other well-trained staff will leave and must be replaced, funding and leadership might change, external systems can change, and new or other social problems arise. Research on the implementation of the same intervention in adult mental health services in Northern Norway showed a significant increase in workers’ identification of COPMI between pre- and post-measurement (22), but only a minor, nonsignificant increase at 5-year follow-up measurement (41). In terms of family conversations, the results indicated that there had not been any significant increase in the employees’ experience with family conversations during the implementation process (2010–2015). These studies demonstrate that even though interventions can be utilized to prevent and reduce the risk that children of patients develop mental illnesses themselves, implementation of new practice is time and resource consuming. In order to succeed in implementing permanent practice changes, sufficient resources such as time, personnel, and training/supervision must be available to the workforce continually (41).

The second reason to interpret our positive result with caution was that changes were not found in all subscales of the questionnaire. One explanation is that the means of the subscales “Support to caregivers and children” and “Coworker support” were already high at pre-test. The scores on these variables were comparable to a sample of Australian Psychiatric nurses (*M* = 5.39, *SD* = 0.84; 39), and hence, the professionals might already give support to caregivers and children and were possibly working together with colleagues on these matters. Another explanation could be that they gave desirable answers before training, as they already knew the importance of including the children of their patients in their regular clinical practice. The whole staff had been informed about the “Semente” program 2 months before training.

The third reason to interpret the results from this study with caution is that the results are based on one measure, a self-report of how the professionals view their family-focused practice. This might not reflect their actual practice, as have been found in other studies (42). Other data should also be collected to find out how many patients with children are identified and in how many cases *Child Talks* is offered (22).

The fourth reason important to acknowledge to be cautious is the lower reliability (α between .44 and .58; see **Table 2**) in 5 of the 10 scales that showed improvement at posttest. We have decided to leave them in because the original scales had satisfactory internal consistency (37). It might be that due to the small sample size of the study, the lower reliability rates were also found in an evaluation study using the questionnaire in a smaller sample (43).

Nevertheless, positive changes took place in the level of knowledge and awareness in the professionals at the hospital, and these changes may provide a solid platform for sustained changes in the focus of clinicians, as well as necessary changes in clinical practice.

### Limitations

This study did not include a control group; this was outside of the scope of the project. Adding a comparison group in a future research might show a clear relation between providing training in COPMI interventions and improvement in family focus practice in the workforce.

Although the FFMHPQ had been proven to be valid and has a reasonable reliability (38), the reliability was poorer in all the subscales in this study. One of the scales (Location issues) had very a low reliability score, Cronbach’s α .26, and was excluded. In addition, the reliability scores of all scales were lower compared to the results of Maybery and colleagues (37). This might be due to the smaller group of subjects in the present study and that the translated version of the scale was not tested for reliability in larger groups in Portugal. We recommend to test the Portuguese questionnaire in a larger sample to make it more reliable in detecting changes in family-focused practice. In the further psychometric development of the measure, Maybery and colleagues (38) report also poor Cronbach alphas in the three scales: Location and engagement issues, and support to carers and children. Future research using the FFMHPQ is necessary to improve the reliability of the weaker items.

Furthermore, the time period between pre and post was only 10 months. This is probably too short to see sustainable practice change. The reason for 10 months post-measurement was due to the lack of project funding after the initial training and implementation. We recommend follow-up measurements to evaluate changes after posttest measures.

Also, as mentioned above, the results are based on self-report; this is a limitation because it does not reflect the actual family-focused practice. More aspects need to be monitored like organizational structure, procedures, and support of the management and also experiences of the families who have received the *Child Talks*.

### Implications and Recommendations

The results from this project indicate that specific training in COPMI interventions may be a way to enable the workforce to move from treatment to prevention and from a patient focus to a family and children focus. Further research with a comparison group is recommended to show that the changes in the professional attitudes are related to training in COPMI interventions. Goodyear and colleagues (27) showed in their study on the workforce in adult mental health care in Australia that practice-based training in family focus was the strongest predictor of family-focused practice. The fact that this program has been developed in a Psychiatric Service is of great relevance to its acceptance and feasibility for the patients, as it is delivered by the community psychiatric team with whom they have a close relationship. Although this is a prevention program for the whole family, it also contributes to reinforce the patient alliance with their mental health professional. The collaboration and the experience of working together in Child and Adult Psychiatry, as well as the regular communication with partners in the community, is interpreted as facilitating factors in the ongoing change process. These factors were prerequisites to the successful provision of support network for families dealing with psychiatry disorders and their children.

Besides introducing *Child Talks* in mental health service supports in the implementation of family focus in the adult health care, further research should evaluate the effectiveness of this brief intervention.

The ethical questions related to the relevance of intervening, as opposed to doing nothing for these children, prompted the professionals who were planning the project to implement a sufficient strategy by including preventive interventions in the mental health care. Given this, we strongly recommend further training of mental health professionals in order to strengthen this new approach from treatment to prevention.

## Ethics Statement

The Ethical Health Commission of Prof. Doutor Fernando Fonseca Hospital approved the project. They determined that written informed consent was not required for this study.

## Author Contributions

All authors contributed to the paper in having read and commented on the manuscript text. KvD, TM, CP, ML, CL, and CR put the paper into writing. TM, CP, ML, JM, and LT collected the data. All authors approved the final version of the paper.

## Conflict of Interest Statement

The authors declare that the research was conducted in the absence of any commercial or financial relationships that could be construed as a potential conflict of interest.

## Footnotes

1 The EEA (European Economic Area) Grants represent the contribution of Iceland, Liechtenstein, and Norway to reducing economic and social disparities and to strengthening bilateral relations with 15 EU countries in Central and Southern Europe and the Baltics.
